# Fertility awareness campaign

**DOI:** 10.5935/1518-0557.20180074

**Published:** 2018

**Authors:** Hitomi Miura Nakagawa

**Affiliations:** 1 GENESIS - Center for Assistance in Human Reproduction, Brasília, DF, Brazil; 2 President of SBRA - Brazilian Society of Assisted Reproduction

There is a worldwide tendency to postpone childbearing. The reasons include personal socioeconomic and cultural improvement, the need to provide for the means and structure to ensure the wellbeing of a baby, life goals such as travelling first and having children later, and waiting for an ideal partner, to name a few (Mills *et al.*, 2011). It is not different in Brazil. Findings from a study published by the Brazilian Institute of Geography and Statistics (IBGE, 2017) showed that the fertility rate in Brazil in 2016 (the mean number of children born by each woman of childbearing age) was 1.73 *versus* 2.44 at a global level (Statista, 2018).

The vast majority of people - including individuals looking for specialized care to have children - believe that assisted reproductive technology can help anyone (Wyndham *et al.*, 2012) with or without reproductive disorders related to gamete quantity and quality and, therefore, tend to refuse treatment options with donor oocytes, sperm or embryos. Although the media often showcases stories of babies born from mothers beyond childbearing age, one should not make wrong assumptions about human natural reproductive performance (Mills *et al.*, 2015).

According to the literature, approximately one in eight couples suffer with infertility (Boivin *et al.*, 2007; Datta *et al.*, 2016). Considering that every person might know someone trying to conceive without success, the Brazilian Society of Assisted Reproduction (SBRA) started a long-term awareness campaign for individuals and governments about reproductive health and behavior, in which the association between age and fertility, natural fertility optimization and preservation, and infertility prevention are discussed.

During the Zika virus epidemics in 2015/2016, the SBRA ran its first large awareness campaign on digital and traditional media - "Gravidez em tempos de Zika" ("Pregnancy in times of Zika") - and partnered with governmental institutions to share accurate information to health care workers and the general population. The Brazilian Society of Infectology (SBI), the Brazilian Society of Urology (SBU), the Brazilian Society of Human Reproduction (SBRH), and the Brazilian Federation of Gynecology and Obstetrics Associations (FEBRASGO) supported the campaign by publishing evidence-based findings and studies.

The year of 2017 revolved around campaign "Fertilidade. O tempo não para" ("Fertility. Time does not stop"), in which the relationship between age and fertility was discussed in scientific meetings and stories on the topic published in the media. Brazil is a country with limited resources and insufficient public services to assist infertile individuals, fraught by long waiting lists and hosts of people losing precious opportunities to complete their families even when they look for assistance at the prime of their reproductive lives.

In 2018, a new interactive campaign was run in ten Brazilian State capitals with the support of local human reproduction stakeholders and health care workers for the "Movimento da Fertilidade" ("Fertility in Motion") program. People were invited to local parks and beaches to engage in physical activities, share their experiences with others, and receive accurate information on the matter. As Norcross (2013) said, the challenge is to make people listen and act based on the knowledge they were given. However, since fertility awareness is low (Harper *et al.*, 2017), education is the way to go.

In the long term, we believe that awareness campaigns and engagement with non-specialized media may help minimize the risk of individuals suffering from infertility due to aging. Social and digital media, along with the press, can be used to further clarify topics in the area of fertility/infertility and shed light on the relevant possibilities offered by assisted reproductive technology.
